# Modified-live PRRSV subtype 1 vaccine UNISTRAIN^®^ PRRS provides a partial clinical and virological protection upon challenge with East European subtype 3 PRRSV strain Lena

**DOI:** 10.1186/s40813-016-0029-y

**Published:** 2016-05-09

**Authors:** Caroline Bonckaert, Karen van der Meulen, Isaac Rodríguez-Ballarà, Rafael Pedrazuela Sanz, Mar Fenech Martinez, Hans J. Nauwynck

**Affiliations:** 1 Laboratory of Virology, Department of Virology, Parasitology and Immunology, Faculty of Veterinary Medicine, Ghent University, Salisburylaan 133, B-9820 Merelbeke, Belgium; 2 Laboratorios Hipra S.A., Amer (Girona), Spain

**Keywords:** Modified-live, Protection, PRRSV, subtype 1, subtype 3 Lena, UNISTRAIN^®^ PRRS, Vaccine

## Abstract

**Background:**

Western European porcine reproductive and respiratory syndrome virus (PRRSV) strains cause limited and mild clinical signs whereas more virulent strains are circulating in Eastern Europe. The emergence of such highly virulent strains in Western Europe might result in severe clinical problems and a financial disaster. In this context, the efficacy of the commercial modified-live PRRSV subtype 1 vaccine UNISTRAIN^®^ PRRS was tested upon challenge with the East European subtype 3 PRRSV strain Lena.

**Results:**

The mean duration of fever was shortened and the number of fever days was significantly lower in vaccinated pigs than in control pigs. Moreover, a lower number of vaccinated animals showed fever, respiratory disorders and conjunctivitis. The mean virus titers in the nasal secretions post challenge (AUC) were significantly lower in the vaccinated group than in the control group. The duration of viremia was slightly shorter (not significantly different) in the vaccinated group as compared to the control group.

**Conclusions:**

Vaccination of pigs with the modified-live vaccine UNISTRAIN^®^ PRRS provides a partial clinical and virological protection against the PRRSV subtype 3 strain Lena.

## Background

Porcine Reproductive and Respiratory Syndrome (PRRS), originally designated Mystery Swine Disease, was first recognized in the United States in the late 1980s and is characterized by late abortion, stillbirth, weak piglets and mummies and is associated with the porcine respiratory disease complex [1]. In 1991, an arterivirus was identified as etiological agent and was scientifically called Porcine Reproductive and Respiratory Syndrome Virus (PRRSV) [2]. During two decades, the virus and its pathogenesis have been studied in-depth, which brought many new insights. There are two main genotypes: the European genotype (genotype 1) and the North American genotype (genotype 2). Three (potentially four) subtypes were already distinguished within the European PRRSV genotype 1 [3]. Subtype 1 is only present in the EU whereas in the Russian area all three (four) subtypes are circulating. After infection with subtype 1, limited clinical signs and respiratory disorders are observed in growing pigs [4, 5]. In contrast, subtypes 2 and 3 are more virulent and infection with subtype 3 strain Lena results in rapid onset of disease with high fever, severe dyspnea and tachypnea, periorbital oedema, depression and mortality [6, 7].

To prevent PRRS, several live-attenuated and inactivated vaccines against PRRSV are commercially available. Attenuated vaccines significantly shorten the viremic phase post challenge [8], but there are concerns on reversion to virulence and the low level of protection upon challenge with heterologous PRRSV strains [9]. Commercial inactivated vaccines are safe, but are not providing a sufficient level of protection [8, 10]. Despite several attempts, no vaccine is providing full protection against the currently circulating PRRSV strains [11]. This might be explained by the low antigenic degree of similarity between the vaccine and challenge strain and the immune evasive character of the virus [12–14]. The co-existence of different subtypes in Europe emphasizes the need for cross-protective vaccines.

Until recently no information was available concerning the efficacy of PRRSV subtype 1 vaccines against PRRSV subtype 3 strains, such as Lena. Surprisingly, a commercially available attenuated subtype 1 vaccine, based on the DV strain, offered partial protection upon the East European strain [15]. This positive result led to the present study, where the clinical and virological protection of another commercially available attenuated subtype 1 vaccine, based on a Spanish PRRSV isolate, was evaluated upon infection with the virulent subtype 3 PRRSV strain Lena.

## Results

### Clinical signs after vaccination and challenge

After vaccination - No adverse local or systemic effects were observed upon vaccination.

**Fig. 1:**
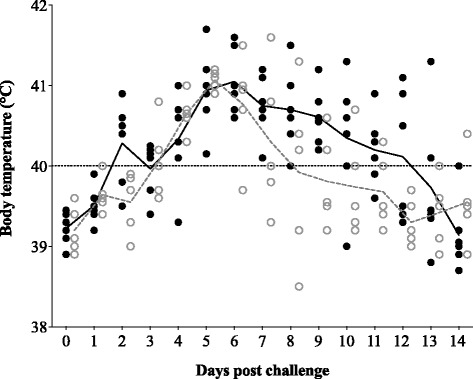
Body temperature after challenge with PRRSV subtype 3 strain Lena. *Bullets* represent individual animals; *lines* represent the mean body temperature in each group. *Solid bullets* and *solid line* show the body temperature for the control group; *open bullets* and *dashed line* show the body temperature for the vaccinated group. *Dotted line* represents the threshold for fever (40.0 °C)

After challenge - The effect of vaccination on body temperature upon challenge is presented in Fig. 1. Overall, a slight beneficial effect of vaccination was observed. In brief, the mean body temperature in control pigs was higher compared to vaccinated animals between 6 and 13 days post challenge (dpc), although the difference was not statistically significant. Also, the area under the curve (AUC) value of fever (with a threshold at 40.0 °C) was higher in the non-vaccinated group (11.7 ± 2.2) than in the vaccinated group (9.7 ± 1.4). A significant beneficial effect was observed for the number of fever days, i.e. the total number of days that an animal showed fever throughout the observation period (9.2 ± 2.9 days for control animals versus 5.2 ± 1.9 days for the vaccinated animals). Also, the number of animals that showed fever per day throughout the observation period was significantly lower in the vaccinated group. Mild respiratory disorders were observed from 2 dpc in non-vaccinated animals and from 5 dpc in vaccinated animals. All animals, except for one unvaccinated pig, showed respiratory signs at least at one time point during the observation period. Respiratory disorders lasted up till 3 weeks post challenge. Scores ranged from 1 to 6 in non-vaccinated animals and from 1 to 4 in vaccinated animals. No significant differences were observed in the mean respiratory score between both groups throughout the study. The AUC value was not significantly higher in the non-vaccinated group (16.1 ± 12.6) than in the vaccinated animals (9.0 ± 9.5). A significant beneficial effect was observed for the number of animals that showed respiratory signs per day throughout the observation period. A slight reduction in liveliness was observed from 2 dpc in non-vaccinated animals and 5 dpc in vaccinated animals and lasted up till 3 weeks post challenge. All animals showed a reduced activity at least at two time points during the observation period and the reduction in liveliness was associated with the occurrence of fever. Discoloration of the ears was regularly observed in all control animals from 6 dpc up till the end of the experiment. In the vaccinated group, mild discoloration of the ears was observed in 4 out of 5 animals between 7 and 17 dpc. Clinical disorders of the eyes, such as conjunctivitis, were seen from 5 dpc in unvaccinated animals and from 6 dpc in vaccinated animals. All non-vaccinated animals showed mild conjunctivitis at least at two time points during the observation period. Only three out of five vaccinated animals showed mild conjunctivitis at least at one time point during the observation period. The AUC value was significantly higher in the non-vaccinated group (3.0 ± 1.7) than in vaccinated animals (0.6 ± 0.7). A significant beneficial effect was also observed for the number of days at which conjunctivitis was observed (6.0 ± 3.3 days for control animals versus 1.2 ± 1.3 days for vaccinated animals). In general, all pigs showed a similar growth rate, independently of vaccination. Mean body weight in the non-vaccinated group at arrival, challenge and euthanasia was 8.1 ± 0.8 kg, 17.9 ± 3.4 kg and 28.8 ± 6.4 kg, respectively. At the same time points, body weight of the animals in the vaccinated group was 8.6 ± 1.0 kg, 15.4 ± 2.7 kg and 27.4 ± 3.9 kg, respectively. At necropsy (28 dpc), macroscopic lung lesions were found in 3/6 control pigs and in 2/5 vaccinated pigs. The total affected lung area in these pigs varied from 0.1 to 0.6 % (control group) and from 0.1 to 2.6 % (vaccinated group). The mean total affected lung area was not significantly different between the groups.

### Serological response upon vaccination and challenge

At the time of arrival (-35 dpc), all pigs were serologically and virologically negative for PRRSV, as determined by immunoperoxidase monolayer assay (IPMA) and virus titration.

**Fig. 2:**
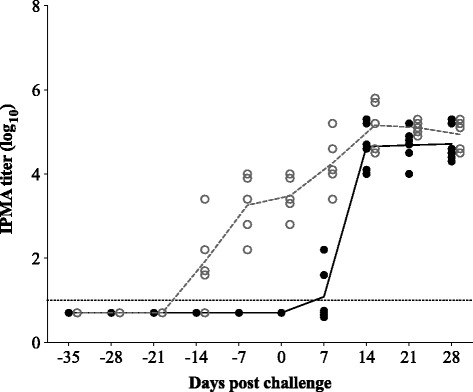
IPMA antibody titers upon vaccination with PRRSV subtype 1 vaccine UNISTRAIN^®^ PRRS and challenge with PRRSV subtype 3 strain Lena. *Bullets* represent individual animals; *lines* represent the mean titer in each group. *Solid bullets* and *solid line* show the titer for the control group; *open bullets* and *dashed line* show the titer for the vaccinated group. *Dotted line* represents the detection limit for the test

**Fig. 3:**
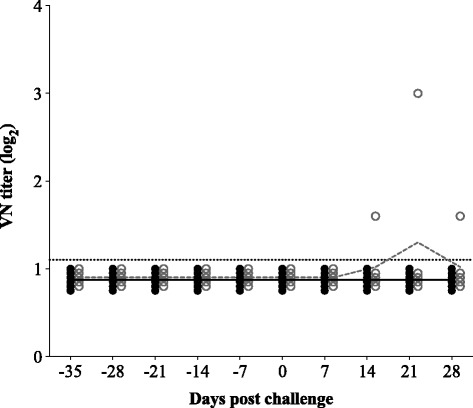
VN antibody titers upon vaccination with PRRSV subtype 1 vaccine UNISTRAIN^®^ PRRS and challenge with PRRSV subtype 3 strain Lena. *Bullets* represent individual animals; *lines* represent the mean titer in each group. *Solid bullets* and *solid line* show the titer for the control group; *open bullets* and *dashed line* show the titer for the vaccinated group. *Dotted line* represents the detection limit for the test

Figure 2 represents the evolution of the IPMA antibody titers during the course of the experiment in vaccinated and unvaccinated control pigs. All control pigs remained seronegative until challenge. The vaccinated pigs seroconverted within two weeks after vaccination with a titer of 3.2 ± 0.8 log_10_ at -7 dpc. After challenge, an increase in IPMA antibody titers was observed in all animals within two weeks. Figure 3 represents the evolution of the virus neutralizing (VN) antibody titers against PRRSV. In both vaccinated and control groups, VN antibodies against PRRSV LV were not detected before challenge. After challenge, VN antibodies against PRRSV Lena were only detected in one out of five vaccinated animals at very low titers (≤3 log_2_) at 21 dpc. A similar pattern in ELISA antibodies was observed as for IPMA antibodies.

### Protective effect of vaccination against viral shedding

**Fig. 4:**
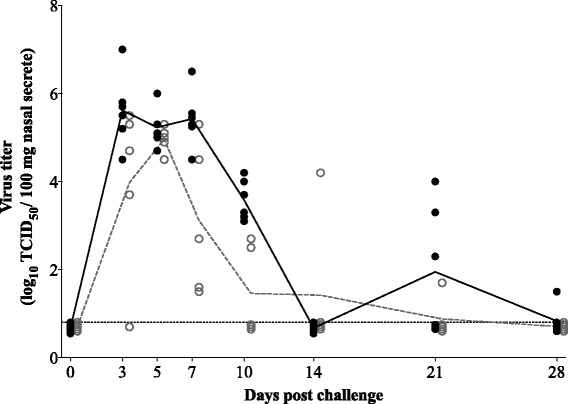
Nasal viral shedding after challenge with PRRSV subtype 3 strain Lena. *Bullets* represent individual animals; *lines* represent the mean titer in each group. *Solid bullets* and *solid line* show the titer for the control group; *open bullets* and *dashed line* show the titer for the vaccinated group. *Dotted line* represents the detection limit for the test

Significant differences were found in viral shedding in nasal secretions. Viral shedding was observed from 3 dpc in both groups (Fig. 4). Titers peaked between 3 and 7 dpc in the control group and at 5 dpc in the vaccinated group. Peak mean titers were 5.6 ± 0.8 log_10_ tissue culture infectious dose with 50 % end point (TCID_50_)/100 mg and 5.0 ± 0.3 log_10_ TCID_50_/100 mg, respectively. In the control group, virus shedding was observed until at least 28 dpc (end of the experiment) with one out of six pigs still shedding virus (1.5 log_10_ TCID_50_/100 mg). In the five vaccinated pigs, viral shedding was observed in all animals up till 10 dpc. After that, two animals shed virus up till 28 dpc. Significant differences in virus titers were found at 3, 7 and 10 dpc. Moreover, the AUC value of virus secretion was significantly lower in the vaccinated pigs (11.6 ± 3.5) than in the non-vaccinated pigs (18.4 ± 1.9).

### Protective effect of vaccination against viremia

**Fig. 5:**
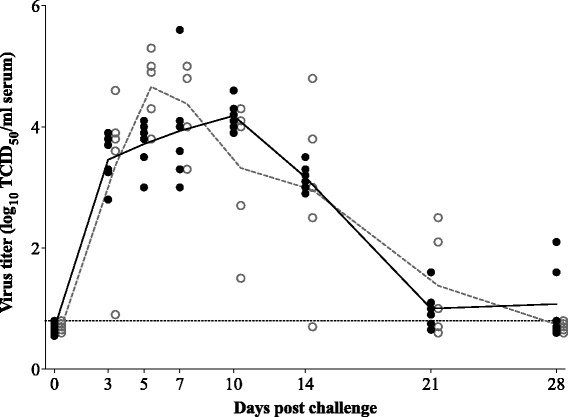
Viremia after challenge with PRRSV subtype 3 strain Lena. *Bullets* represent individual animals; *lines* represent the mean titer in each group. *Solid bullets* and *solid line* show the titer for the control group; *open bullets* and *dashed line* show the titer for the vaccinated group. *Dotted line* represents the detection limit for the test

The results of virus titrations of sera (viremia) are shown in Fig. 5. Virus was present in sera of all animals from 3 dpc. A peak was observed at 10 dpc in the control group (4.2 ± 0.2 log_10_ TCID_50_/ml) and at 5 dpc in the vaccinated group (4.7 ± 0.6 log_10_ TCID_50_/ml). In the control group, viremia lasted until at least 28 dpc (end of the experiment) with two out of six piglets still being viremic, although at low titers (2.1 and 1.6 log_10_ TCID_50_/ml). In the vaccinated group, viremia lasted until 21 dpc. Despite the shorter duration of viremia, no significant differences were observed in the mean virus titer between control and vaccinated pigs. Also, no significant differences were seen for AUC values between both groups (15.4 ± 0.8 in control animals versus 15.9 ± 2.0 in vaccinated animals).

## Discussion

Reproductive failure with early farrowing, late abortion, still- and weakborn piglets in sows and infertility in boars on the one hand and respiratory disorders in piglets on the other hand are hallmarks of PRRS. Depending on the strain genotype, host genotype and co-infections, divergent clinical signs can be observed in piglets. Pigs are well protected against a challenge or re-infection with a homologous strain after a natural infection [8, 12, 16, 17]. This can be mimicked using modified-live vaccines [18, 19]. However, after a heterologous challenge different levels of protection can be obtained [16, 18–20]. In general, animals are partially protected, both clinically and virologically. Labarque et al. found evidence that a genetic diversity within European strains of subtype 1 affects the efficacy of European vaccines and similar findings were described after natural exposure within the same subtype [12, 16]. In the present study, the efficacy of a vaccination with UNISTRAIN^®^ PRRS (genotype 1, subtype 1) was examined upon a challenge with PRRSV strain Lena (genotype 1, subtype 3). The mean duration of fever was shortened and the number of fever days was significantly reduced in vaccinated pigs compared to unvaccinated control pigs. Scores for respiratory and eye disorders were assigned to fewer vaccinated animals than to control pigs. Based on these results, the vaccine is considered to raise an immunity that gives a partial clinical protection against heterologous infection. In addition, the vaccination with UNISTRAIN^®^ PRRS offers also a partial virological protection. Significant differences in mean nasal PRRSV titers were observed at 3, 7 and 10 dpc and titers were reduced with 1.64, 2.29 and 2.14 log_10_ TCID_50_/100 mg, respectively. In addition, the total nasal viral shedding (AUC) upon challenge was significantly lowered with a factor 6.8. Similar findings concerning nasal secretion after vaccination with a commercially available live-attenuated vaccine based on the DV strain upon challenge with PRRSV Lena were recently described [15]. The high titers in nasal secretions in control animals might have influenced the process of viral shedding since transmission through viral shedding is considered to be an efficient way of re-infecting pen mates and is a measure of safety and efficacy of commercially available vaccines. In present study, a sudden drop in PRRSV-titer in nasal secretions is observed at 14 dpc in control animals, after which three control pigs secrete the virus at 21 dpc. In the vaccinated group, one piglet shed PRRSV again at 21 dpc. The ratio of 3 re-infected control pigs and 1 re-infected vaccinated piglet suggest the protective effect of vaccination, which is in agreement with previous studies [21, 22], although this experimental design did not allow us to determine the transmission ratio. Despite the positive outcome for nasal shedding, vaccination with UNISTRAIN^®^ PRRS only slightly reduced the duration of viremia. In the study of Trus et al. [15], the viremia was significantly reduced upon challenge with PRRSV strain Lena in pigs vaccinated with a commercially available live-attenuated vaccine based on the DV strain. This difference in protection might be explained by different factors, such as age and breed of the pigs, interval between vaccination and challenge, vaccine and challenge virus titer and antigenic homology between the vaccine strain and challenge strain. Although the regions that are responsible for the induction of neutralizing antibodies and cellular immunity have been identified [23–28], there is no clear correlation between genetic homology and antigenic homology [12, 13, 15, 29]. Therefore, it is difficult to estimate the impact of slight genetic differences on the immunogenicity of the vaccine virus and the protection upon challenge (PRRSV strains DV and Lena have an identity of 88 % whereas VP-046 BIS and Lena have an 82.5 % identity (ORF5)). The different outcomes between both studies cannot be related to the genetic background in our opinion, as the piglets came from the same farm [30, 31]. The major difference between both studies was the vaccination-challenge interval. In the studies described by Trus et al. [15], the interval was 6 and 8 weeks, which is two and four weeks longer than in the present experiment. After infection and vaccination with PRRSV, the immunity is slowly induced and it has been shown that protection six or eight weeks after vaccination is better than after four weeks [14].

The virus neutralizing (VN) antibodies have a crucial role in prevention of disease caused by Equine Arteritis Virus (EAV) in horses [32] and Lactate dehydrogenase-elevating virus (LDV) in mice [33]. Similarly, inhibition of the PRRSV replication can be achieved by VN antibodies [34]. However, neutralizing antibodies appear late after PRRSV infection [35] or vaccination with modified-live PRRSV vaccines [8, 36]. Thus, it is not surprisingly that in the present study no VN antibodies were detected during the four-week vaccination-challenge interval.

After a homologous challenge, the VN antibodies against the challenge virus are boosted [8, 15, 36, 37] in contrast with a genotypically heterologous challenge where no VN antibodies against the challenge virus are detected [38, 39]. After challenge with the subtype 3 strain Lena, VN antibodies against Lena were only detected in one UNISTRAIN^®^ PRRS-vaccinated animal during 2 weeks. In a similar experiment using another PRRSV subtype 1 vaccine, based on the DV strain, the pigs developed VN antibodies against Lena 1-2 weeks after a PRRSV Lena challenge. In this latter study, the viremia was clearly more reduced compared to their non-vaccinated control group, which might be explained in part by the presence of the neutralizing antibodies. However, in the present study, the pig that developed VN antibodies against Lena did not show a shorter duration of viremia or nasal shedding. The role of VN antibodies in protection is therefore again disputable [40]. Certain branches of the cell-mediated immunity are most likely more important and can be assessed by measuring interferon gamma (IFN-γ) producing cells [41]. During this study, a test to determine the levels of the IFN-γ was not available in our laboratory and was therefore not assessed. We do agree that those results could have given an extra value and are implementing this technique in current studies.

## Conclusions

The present study demonstrates that vaccination with the modified-live vaccine UNISTRAIN^®^ PRRS provides a partial clinical and virological protection upon challenge with PRRSV Lena. Because only a partial clinical and virological protection has been obtained with currently commercially available subtype 1 PRRSV vaccines, there is a need to design vaccines that give a better protection against PRRSV Lena.

## Methods

### Experimental design

Eleven piglets were purchased from a PRRSV-negative farm immediately after weaning. Their negative PRRSV status was confirmed by serology (IPMA) and by virus titration of sera and nasal secretions that were collected upon arrival. They were acclimatized during seven days after which the animals were randomly assigned to two groups. One group (*n* = 5) was vaccinated intramuscularly with 2 ml of the commercially available live attenuated PRRSV subtype 1 vaccine (UNISTRAIN^®^ PRRS, Laboratorios Hipra S.A.). Retitration revealed a titer of 6.8 log_10_ TCID_50_ per ml. The second group was mock-vaccinated with phosphate buffered saline (PBS) (*n* = 6). At 4 weeks post vaccination, all pigs were intranasally inoculated with 2 ml of 5 log_10_ TCID_50_ PRRS virus strain Lena (subtype 3) [6]. Blood was collected on a weekly base to monitor the serological status (IPMA and VN). To follow the course of viremia and nasal shedding upon challenge, blood and nasal swabs were collected on 0, 3, 5, 7, 10, 14, 21 and 28 dpc. At 4 weeks post challenge, the experiment was terminated by intravenous injection of an overdose of sodium pentobarbital (Natrium pentobarbital 20 %, Kela Laboratoria nv, Hoogstraten, Belgium).

### Serology

Fixed PRRSV Lelystad virus (LV) respectively Lena infected Marc-145 cells in 96-well microtiter plates were used for the IPMA [4]. Serial twofold dilutions of the serum samples were added to the plates and incubated for 1 hour at 37 °C. After washing, secondary goat anti-swine IgG labeled with peroxidase were added for another hour at 37 °C. Plates were washed again and a substrate solution of 3-amino-9-ethylcarbazole (AEC) was added to each well, followed by incubation of the plates at room temperature for 20 minutes. The IPMA titer is the reciprocal of the highest dilution that gives a coloration of infected cells. VN antibodies were detected by SN assays in Marc-145 cells using PRRSV LV in sera collected before challenge and PRRSV Lena in sera collected after challenge. Twofold dilutions of serum samples were prepared and 100 μl of the appropriate PRRSV strain with a titer of 2 log_10_ TCID_50_/50 μl was added. After mixing, the plates were incubated at 37 °C for 1 hour and 50 μl of the mixture was subsequently transferred to confluent monolayers of Marc-145 cells in 96-well microtiter plates. Cells were screened for 7 days after inoculation and the neutralization titer of the sera was recorded as the reciprocal of the highest dilution that inhibited CPE in 50 % of the inoculated wells. Additionally, sample to positive ratios were determined using the CIVTEST SUIS PRRS E/S^®^ ELISA (Laboratorios Hipra S.A.) with the aim to detect antibodies against European PRRSV isolates. The ELISA was performed according to the manufacturer’s instructions.

### Evaluation of clinical signs

Body weight was monitored for all pigs upon arrival (-35 dpc), at challenge (0 dpc) and at euthanasia (28 dpc). Local side effects as well as body temperature were recorded at 1, 2 and 3 days post vaccination (dpv). After challenge, the animals were monitored daily for the presence of clinical signs up till day 14 post challenge, with particular attention to PRRS related clinical signs. Clinical parameters included body temperature, respiratory symptoms, liveliness, discoloration of the ears, clinical symptoms at the eyes and presence of diarrhea. A score was assigned to the various parameters to allow an objective comparison between both groups. The scores were based on the methodology described by Karniychuk et al. [6] and Weesendorp et al. [7]. Lungs were collected and macroscopic lung lesions were given a score by visual observation and computer-assisted analysis. The percentage of lung surface affected by pneumonia was estimated by multiplying the lung lesion score per lobe with the relative proportion of this lobe in the entire lung [42].

### Virus titrations

At 0, 3, 5, 7, 10, 14, 21 and 28 dpc, serum was tested virologically (titration) to follow the course of viremia. In addition, nasal secretions were collected with dry swabs (COPAN 160C^®^), 1 ml transport medium (phosphate buffered saline supplemented with antibiotics and fetal calf serum) was added and the swabs were vortexed and centrifuged. Supernatant was used for virus titration. In brief, porcine alveolar macrophages (PAM) were cultivated for 24 hours and inoculated with 10-fold dilutions of either serum or nasal secretion. After 72 hours, cells were fixed and virus-infected cells were subsequently evaluated by subsequent incubation with PRRSV-specific monoclonal antibodies against the nucleocapsid protein.

### Ethics statement

The study was conducted in compliance with the provisions of Directive 86/609/EEC and KB 29/05/2013 and received approval number EC 2013/157.

### Statistics

Data were analyzed with GraphPad Prism 6 software (GraphPad Software Inc., San Diego, CA, USA). All results shown represent mean ± standard deviation (S.D.) or, for IPMA titers, geometric mean value ± S.D. Serological titers (IPMA and VN), as well as viral loads, were log-transformed prior to analysis. Gross pathology scores and area under the curve (AUC) were analyzed using the non-parametric Mann Whitney test. Duration was evaluated by the t-test. Statistical analysis of continuous data was performed using repeated-measures two-way analysis of variance (rANOVA), with Bonferroni’s post-test. Results with *P*-values ≤ 0.05 were considered statistically significant.
